# Are Antibiotics the New Appendectomy?

**DOI:** 10.7759/cureus.44506

**Published:** 2023-09-01

**Authors:** Janan Alajaimi, Manar Almansoor, Amina Almutawa, Maryam M Almusalam, Husham Bakry

**Affiliations:** 1 School of Medicine, Royal College of Surgeons in Ireland, Busaiteen, BHR; 2 Surgery, King Hamad University Hospital, Busaiteen, BHR; 3 General Surgery, King Hamad University Hospital, Busaiteen, BHR

**Keywords:** conservative vs surgical management, appendectomy, antibiotics, uncomplicated appendicitis, complicated appendicitis, appendicitis

## Abstract

Prior to the development of laparoscopic procedures, open appendectomy was the standard of care for the majority of appendicitis cases. Recently, studies have debated using antibiotics as a first-line treatment in uncomplicated appendicitis cases. The definition of uncomplicated appendicitis is not always clear-cut; however, with the large-scale accessibility of radiologic techniques, it is becoming increasingly easier to classify patient groups. As suggested by clinical and radiological patient data, this has raised the speculation of considering antibiotic therapy as the sole treatment modality in uncomplicated appendicitis cases. We aim to compare the options of surgery and antibiotics only in terms of efficacy, complications, and financial cost.

A range of databases and search strategies were adopted, and various databases were used, including PubMed, ScienceDirect, Google Scholar, and JAMA. Collectively, 30 studies were reviewed, but only 18 were included.

Efficacy rates were higher in the appendectomy group. Nevertheless, the antibiotics-only group maintained an efficacy rate greater than 70% at one-year follow-up. Risk factors that decreased the efficacy in medical management included the presence of appendicolith, neoplasm, appendiceal dilatation, peri-appendiceal fluid collection, higher mean temperature, CRP, and bilirubin. Complications were more frequent and significant in the surgery group. These included complications related to anaesthesia, surgical site infections, damage to nearby structures, and pulmonary embolism. Despite several years of follow-up and disease recurrences, higher financial costs were observed in surgically treated patients compared to the antibiotics-only group.

Given the high success rates post-appendectomy for acute appendicitis over the decades, the efficacy of conservatively treated acute appendicitis raises a strong argument when choosing one of the two options. The efficacy remained consistently higher across the literature in the surgery group than in the antibiotics-only group. However, it is still arguable that antibiotics may be a preferable option given an efficacy rate of more than 70% at one year and overall higher complications associated with surgery. The argument of missing a neoplasm by avoiding surgery is valid. However, most are carcinoid neuroendocrine neoplasms with a low probability of metastasis (<5%) and are usually considered benign. Given the current practice focused on conservative and minimally invasive treatments and recently the COVID-19 pandemic, with its restrictions and lessons learnt, antibiotics may be the future standard for treating uncomplicated acute appendicitis. Lastly, we noticed higher efficacy rates in articles published recently than those published at least five to ten years earlier.

Antibiotics-only therapy for uncomplicated appendicitis is cost-effective with fewer complications than surgery. However, appendectomies have higher efficacy. Thus, surgical treatment prevails as the standard of care. Future literature should yield larger sample sizes and explore the numbers of emergency appendectomies mandated following antibiotics-only therapy.

## Introduction and background

Background and importance

More than a century ago, appendectomy was the standard of care for all appendicitis patients, despite recent epidemiological and clinical data indicating that there may be two distinct types of acute appendicitis. Uncomplicated and complicated acute appendicitis, two kinds of disease with varying severity, seem to be separate entities rather than successive occurrences [1]. Complicated acute appendicitis, often described as a finding of perforation, abscess, or a suspicion of a tumour, necessitates prompt surgical treatment [2-4]. However, the management of uncomplicated appendicitis has been an area of contention.

Since McBurney first described the condition in 1889, open appendicectomy has been the primary method of treating acute appendicitis. It is also widely believed that appendicitis frequently develops from simple to perforated appendicitis without surgery [5].

Numerous studies have established laparoscopic appendectomy as the current gold standard treatment for appendicitis as it explicates better results than open appendectomy due to substantial developments in laparoscopic techniques and instruments [6]. According to a meta-analysis of 33 prospective randomised controlled trials that included more than 3500 patients, laparoscopic appendectomy in adults was associated with a statistically significant reduction in the incidence of length of hospitalisation, post-operative complications, and recovery time [6,7].

Antibiotic therapy was frequently recommended as a bridge before surgery in patients with suspected appendicitis with no apparent causes for appendicectomy, such as evidence of perforation or peritonitis. However, due to inherent pitfalls in the quality and design of individual trials, the routine use of antibiotics in patients with uncomplicated acute appendicitis was not adequately supported. Given that other intra-abdominal inflammatory diseases, including colonic diverticulitis, are frequently treated non-operatively, the significance of antibiotic therapy in acute uncomplicated appendicitis may have been underestimated solely based on tradition rather than evidence [5]. It is possible to identify a minor proportion of cases with complex appendicitis owing to the availability of diagnostic modalities, including computed tomography (CT) and ultrasound (US). Furthermore, epidemiological research indicates that, despite a rising tendency in surgical exploration for suspected appendicitis over the years, the incidence of perforated appendicitis has remained consistent across all age groups [5].

Despite these findings, most patients with uncomplicated acute appendicitis worldwide still undergo appendectomy. In the USA, only 6% of patients with uncomplicated appendicitis receive conservative treatment with antibiotics; the majority of patients undergo laparoscopic appendectomy [8].

The need for an immediate appendectomy for all patients with uncomplicated acute appendicitis may be further reconsidered in light of these findings [9]. As a result, a thorough evaluation of the best treatment option should also take into account outcomes unrelated to the compared treatment strategies, such as treatment-related morbidity, recovery time, and post-intervention pain, as well as patient-related factors like the patient's preference in light of the patient's current situation and treatment costs.

Goals of this review

The advent of laparoscopic appendectomy has replaced open appendectomy in previous years, so should the non-operative management of appendicitis be the new standard of care in the current era? This review aims to compare antibiotic treatment with appendectomy for uncomplicated acute appendicitis in the adult population, particularly regarding safety, efficacy, and cost analysis.

We conducted this review to describe, compare and summarise the published literature about operative versus non-operative acute uncomplicated appendicitis management and to summarise and highlight the best practices to implement in managing acute uncomplicated appendicitis with the goal of finding the current gaps in knowledge to further base further trials on.

Methods

Search Strategy and Selection Criteria

**Figure 1:**
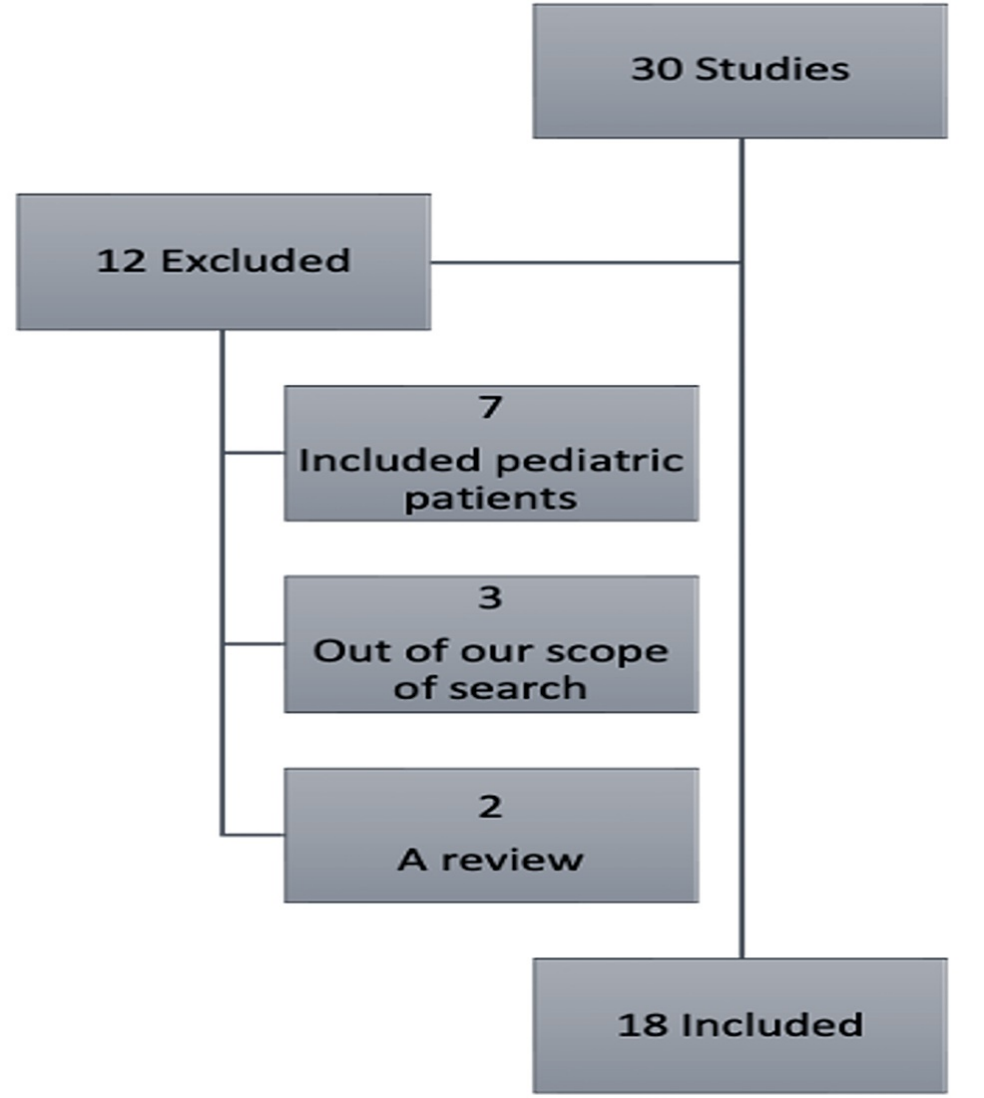
A flowchart of the selected studies

A range of databases and search strategies were adopted to create a comprehensive overview that addresses the topic from various angles. PubMed, ScienceDirect, Google Scholar, and JAMA were mainly searched from July 2022 to August 2022. Papers were only referenced if they were primarily written in English or secondarily referenced in English from a different language. A diverse list of works was reviewed and discussed, ranging from case reports to systematic reviews. Selected studies comparing antibiotic treatment with appendectomy for uncomplicated acute appendicitis in adult patients were eligible for inclusion. We included studies with well-defined diagnostic and treatment protocols. We excluded studies with the pediatric population, review papers, and studies that reported outcomes in patients with complicated appendicitis (local or contained perforation with an appendicular abscess or mass). We found 30 studies, and only 18 met the inclusion criteria and thus were included in our review, as shown in Figure 1.

## Review

Efficacy of conservatively treated acute appendicitis

Out of all the studies reviewed, the efficacy of conservatively treated acute appendicitis was 70.62% and 60.95% at one- and five-year follow-ups, respectively.

In a randomised controlled trial (RCT) including 1,552 adults, 776 in each arm (surgery versus antibiotics only), patients in the antibiotic group received at least 24 hours of intravenous antibiotic therapy followed by oral antibiotic treatment for 10 days. Of these patients, 11% converted to surgery by 48 hours, 20% by thirty days, and 29% by 90 days. Moreover, at least one additional course of antibiotics was prescribed within 90 days after the primary treatment in 11% of patients in the antibiotics group. The percentages of emergency department visits after primary treatment were 4% in the appendectomy group and 9% in the antibiotics group [10].

Moreover, in a prospective multicenter study involving 318 patients, 87 patients in each arm as the matched group, 73.6% of patients in the antibiotics group did not require an appendectomy at one year. Also, an additional course of antibiotics successfully treated 45.5% of patients with recurrences after primary treatment. This highlights the fact that the efficacy of antibiotic therapy decreases in a recurrent episode [8]. 

Furthermore, an RCT including 530 adult patients found that 61% of patients in the antibiotic group did not undergo an appendectomy at five years. It was also detected that most recurrences occurred within the first year after primary treatment, with a marked decline in recurrences after the second year. The cumulative recurrence of acute appendicitis was reported annually for the first five years, 27.3% at one year, 34% at two years, 35.2% at three years, 37.1% at four years, and 39.1% at five years [11]. 

The APPAC is an RCT completed in 2018 following five years of follow-up. It included 530 patients, with 256 patients in the antibiotics group. At one year, 70 patients (27.3%) underwent an appendectomy, with 5.8% converting to surgery at the initial hospitalisation. Additionally, 30 cases required an appendectomy between one and five years. The cumulative recurrence rate was 34% at two years, 35.2% at three years, 37.1% at four years, and 39.1% at five years [3,12]. 

Furthermore, a systematic review and meta-analysis of five RCTs, including 1351 patients, 632 in the antibiotics group and 719 in the appendectomy group, revealed a higher efficacy in the appendectomy group based on a one-year follow-up. The effectiveness at one year was 98.3% and 75.9% in the appendectomy and antibiotics groups, respectively [13]. In two other reviews, one of which included 2751 patients, an efficacy of 82.8% has been reported in the antibiotics-only group. Alternatively, an efficacy of 70% has been delineated in the other [14,15]. In a clinical trial with 119 patients, 7.6% failed to respond to primary antibiotic treatment, requiring conversion to surgery. Over a median follow-up period of 14 months, 12.7% experienced a recurrence. The study concluded that in about 80% of patients, primary treatment with antibiotics alone was successful, thus avoiding the need for appendectomy. However, this study was limited to patients with an appendiceal diameter of ≤10mm [16].

Three studies shared similar success rates over a follow-up period of one year, in which most recurrences tend to occur in patients with conservatively treated acute appendicitis [17,18,19]. Gandy et al., Sallinen et al., and O'Leary et al. reported success rates of 77%, 76.2%, and 74.7%, respectively, one-year following primary treatment. Conversely, five other studies published five to 10 years earlier show lower success rates than the aforementioned [5,20-23]

In 2010, Shindoh et al. reported a success rate of 59.4% for those primarily treated with antibiotics. Shindoh et al. concluded that despite the low success rate, surgery might be avoided initially with antibiotics without increasing morbidity [21]. Within the same year, Varadhan et al. demonstrated a similar success rate of 57.14% at one-year follow-up based on a systematic review and meta-analysis including 350 patients [22]. In their primary admission, 68% were treated successfully with antibiotics, with the rest crossing over to surgery. During the one-year follow-up, 15% of patients experienced a recurrence. About 8% of those recurrences have retreated successfully with antibiotics. Varadhan et al. concluded that antibiotics were unlikely to replace appendectomies 13 years ago. Varadhan et al. in another systematic review and meta-analysis published two years later, with a total of 900 patients reported a success rate of 63% at one-year follow-up [22]. Varadhan et al. concluded that uncomplicated acute appendicitis might be treated primarily with antibiotics, similar to uncomplicated acute diverticulitis. Vons et al. had similar success rates to Varadhan et al., with 63.3% not requiring surgery at one year. The study included 239 patients, with 14% requiring surgery at one month, rising cumulatively to 36.7% at one year [5].

In 2011, Ansaloni et al. compared treatment efficacy between those primarily treated with surgery or antibiotics, giving a pooled estimate of 90.1% and 57.9%, respectively [20]. Ansaloni et al. were not certain about antibiotics as viable surgical substitutes, elaborating that additional, high-quality RCTs are required for further investigation. Furthermore, although these studies were published during the same time, they had proven higher success rates compared to those mentioned above [24,25]. Liu et al. included 1,201 patients in their analysis, 6.9% of whom failed to respond to primary antibiotic treatment and required surgery [24]. Additionally, 14.2% experienced a recurrence, giving an overall efficacy of 78.9%. Paajanen et al. started the APPAC trial to support the hypothesis that 75-85% of patients with uncomplicated acute appendicitis can avoid surgery [25]. Results of the interim analysis in 2013, including 161 patients, correlated with the study's hypothesis.

Risk factors for conversion to appendectomy and recurrence following conservatively treated acute appendicitis

The risk factors associated with conversion to appendectomy and recurrence in patients treated conservatively with antibiotics as first-line management were found partly related across the studies reviewed.

Davidson et al. concluded that participants with an appendicolith were at a higher risk for conversion to appendectomy. The possibility of a neoplasm was also thought to increase the risk of conservative management and surgery failure. In this study, neoplasms were identified in nine patients, seven of whom were already allocated to the appendectomy group, and two converted from the antibiotics group to surgery [10].

A review article suggested that specific CT findings may predict patients in whom antibiotics, as first-line treatment, may fail. High-risk CT findings were defined as the presence of an appendicolith, mass effect, and an appendiceal diameter ≥13mm. Surgical management is recommended in this cohort instead of antibiotics only as first-line treatment [14]. Another review article found that a higher mean temperature was the only significant factor that predicted the crossover of patients from antibiotic-only treatment to appendectomy, with no differences in laboratory test results or clinical examination findings. Participants in the crossover group had an overall higher mean temperature of 37.5° C compared to 37.2° C in participants who did not require crossover to appendectomy [18].

Moreover, an original article also considered the presence of an appendicolith in imaging studies and an elevated CRP (>4mg/dL) as significant factors for conversion to appendectomy and as general predictors of a negative outcome in the non-operative management of acute appendicitis. Furthermore, the maximum diameter of the appendix, the incidence of pericycle fluid collection, and the presence of calcified appendicolith showed significant differences between the two groups [23].

Morbidity and mortality of conservatively versus surgically treated acute appendicitis

Various studies focus on major and minor complications of appendectomies at a more significant percentage than antibiotics [8,12,13,18,19,26]. The types of complications in most studies may also be classified into immediate, early, and late, as not all the studies have classified operative complications into major and minor. Anaesthesia-related complications were also mentioned, but not in great depth. Intra-operative complications mainly included bleeding, damage to nearby structures such as the urinary tract and bowel, and vascular damage. Post-operative complications may be subdivided into early and late. Early complications mainly include hematoma formation, fistulae, abscesses, surgical site infections (SSIs), adhesions, and eventually obstruction. Late complications included hernia, adhesions, obstruction, and tubal infertility [26].

Irrespective of their order, the most reported complications are bowel obstruction due to mechanical and non-mechanical causes, post-operative abscess, peritonitis and perforation, and SSIs [3,8,12,18,26,27]. According to studies that recorded such complications, the incidence of these conditions ranged from 3% [28] to 24.4% within five years of follow-up [9]. In another study, complications may reach up to 28% [29]. On a serious note, cardiac and pulmonary conditions such as pulmonary embolism (PE) accounted for 0.6% of the complications in some instances [13,18,27].

The incidence of removing a normal appendix has not been mentioned in the studies included in this paper. However, a study in the United Kingdom, including 5,345 appendicitis cases, reported that normal appendectomies were seen in 28.2% of females and 12% of males [28]. Considering these percentages, in 2016 Gandy et al. noted that removing a normal appendix could increase surgical complications by 17% [17]. Earlier in 2011, a study recognised that the practice of urgent appendectomies increased surgical complications by 35% [24]. Nonetheless, emergency appendectomies carry the most significant risk as opposed to elective cases [15]. 

In terms of morbidity and mortality, operating on patients with CT findings of fecalith, dilation, and mass effect would have low post-operative morbidity and mortality [14]. Conversely, a meta-analysis in 2019 concurs with the low surgical mortality; however, it describes higher post-operative morbidity [19]. On the contrary, a 2014 study reported opposite findings and concluded that a higher morbidity rate was appreciated in the antibiotic group [30].

As mentioned above, higher complications reported in negative appendectomies carry a risk of 0.02% mortality [18] and a 30-day mortality rate of 0.4% [3]. Generally, studies describe a higher morbidity rate that reaches up to 3.5 times in surgically intervened cases [18,25]. Inversely, only one study has discussed the possibility that patients treated with antibiotics only may have a lower morbidity and mortality rate [24]. 

Another complication, although relatively rare, is missed cases of appendiceal neoplasms. This has occurred in 1.5% of the patients in a study, with the commonest subtype being neuroendocrine carcinoid tumours [3]. 

Financial costs

Comparing the financial costs of uncomplicated appendicitis treated with antibiotics only versus surgery is a weakly tackled factor in the literature reviewed [11]. However, some studies analyse this while comparing the two variables. 

For instance, Park et al. illustrated higher medical costs in patients treated surgically compared to those with antibiotics only, with a difference that reached $1067 as presented in Table 1 [16].

**Table 1:** A cost comparison between patient groups treated with surgery versus antibiotics only

	Medical Cost Difference	Productive Cost Difference	Total Cost Difference
The APPAC Trial (One-year follow-up) [16]	$1,010	$1,117	$2,127
Park et al. [17]	$1,067		$1,067
The COMMA Trial [18]	$1,862		$1,862

Furthermore, the APPAC trial shared a similar conclusion to that reported by Park et al. depicting higher costs in surgically treated patients compared to the antibiotics-only group at one- and five-year follow-ups, where the total costs were 1.6 and 1.4 times higher, respectively. The change in cost at one and five years is due to the elevation in their hospital charges after the appendectomies performed post-appendicitis recurrence [11]. Nevertheless, this study calculated the total costs more specifically as it translated the days stayed at the hospital and the sick leaves on discharge into productivity losses, which measures the costs unrelated to the medical treatment. Haijanen et al. calculated the per-day productivity costs by dividing Finnish's average monthly gross wage based on sex and by the number of working days, which is 21 [11]. Moreover, sensitivity analysis was used to get less biased costs.

Additionally, the COMMA trial also tackled the cost factor in their study. Their results expressed a reduced cost in the antibiotic-only group compared to the surgically treated patients with a difference of $1,862, as explained in Table 1 [17]. On the other hand, the mean cost of the patients who had appendicitis recurrence and underwent appendectomy later on was higher than the mean cost of patients who went through appendectomy initially, with an $889 difference. This is due to the readmission and surgical expenses [17].

Nevertheless, the studies above had some limitations in their cost analysis. Firstly, Park et al. and the COMMA trial did not consider the productivity losses when counting their total costs [16]. Secondly, both Park et al. and the APPAC trial did not illustrate the expenses of the recurrence group treated patients' costs alone. This is a notable figure for organisations and patients contributing to their decision to adopt a management plan. Thirdly, the COMMA trial analysis excluded patients who previously had an anaphylactic reaction to penicillin [17], a significant proportion of the patients encountered in a clinical setting. Fourthly, all three studies did not consider Clostridium difficile infections in their cost analysis. Lastly, the trials did not include the productivity losses related to medical leaves given to caregivers.

Discussion

Given the higher success rates post-appendectomy over the decades, the lower efficacy of conservatively treated acute appendicitis raises a strong argument when choosing one of the two options. The efficacy remained consistently higher across the literature in the surgery group than in the antibiotics-only group. However, it is still arguable that antibiotics may be a reasonable option given an efficacy rate of more than 70% at one year, especially with the overall higher complication rate associated with surgery. Given the inconclusiveness of the results, like other things in clinical medicine, a decision must be taken case by case. Thus, different factors should come into play when choosing between antibiotics versus surgery when treating uncomplicated acute appendicitis, instead of only leaving antibiotics a feasible option when surgery is not one, e.g., in elderly patients with multiple comorbidities.

Although antibiotics-only may be primarily efficacious in treating uncomplicated acute appendicitis, some develop recurrences [31]. Furthermore, the antibiotics-only approach may be re-attempted in these cohorts. Despite that, it had worked in almost 50% of those with recurrences as per Podda et al [8]. These multiple courses of antibiotics can add to an already existing issue of antimicrobial resistance, which is thought to be a pandemic in the shadows [32].

The risk factors that increased the risk for conversion to appendectomy and/or recurrence were the presence of an appendicolith with or without calcifications, neoplasm, appendiceal dilatation, peri appendiceal fluid collection, higher mean temperature, and high CRP. Thus, we believe that patients with any of these characteristics should have a lower threshold for surgery.

Conversely and cautiously, antibiotics-only may be the approach in patients with none of the above-mentioned characteristics under close monitoring and after careful consideration of other aspects of their clinical status, including but not limited to their symptoms and physical exam findings.

An integral part of patient-centred care and modern medicine is involving the patient in their own management plan by allowing them to make an informed decision based on findings in the literature. Thus, the choice of surgery versus antibiotics can come down to individual patients and their preferences. Many patients are willing to opt for surgical management despite the complications and additional financial costs that come with it, as it is more definitive. Contrastingly, some would want to avoid surgery whenever possible and accept the chances of recurrences and the potential need for surgery later.

The argument of missing a neoplasm by avoiding surgery is valid. However, the majority are carcinoid neuroendocrine neoplasms with a low probability of metastasis (<5%) and are usually considered benign [33].

The financial cost is one of the deciding factors for patients, doctors, and institutions when making a clinical decision. Most studies reported higher financial costs in terms of, but not limited to, days stayed at the hospital and sick days in the appendectomy group than in the antibiotic group. Furthermore, the APPAC trial added that despite the recurrences in the antibiotic group requiring appendectomies later, the costs were still lower in those treated with antibiotics initially [11]. Alternatively, the COMMA trial suggested otherwise, with higher costs in those initially treated with antibiotics due to the recurrences that ultimately require appendectomies [17]. That said, more trials should include cost analysis in their research when comparing antibiotics-only and surgery in treating uncomplicated appendicitis.

During the COVID-19 pandemic, particularly during the outbreak between March and April 2020, a retrospective audit showed a significant decrease in emergency surgery admissions with only slight changes in single diagnoses and treatments. These findings open doors of opportunities for different ways surgical emergencies, including acute appendicitis, may be managed without overcrowding hospitals [34]. Given the current practice focused on conservative and minimally invasive treatments and recently the COVID-19 pandemic, with its restrictions and lessons learnt, antibiotics may be the future standard for treating uncomplicated acute appendicitis.

We believe that future research in this field should be directed towards knowing how many of those with recurrences following conservative management required emergency versus elective appendectomies, as that would raise another vital point that may either make or break the argument of conservatively treating uncomplicated acute appendicitis.

Lastly, we noticed higher efficacy rates in articles published recently than those published at least five to 10 years earlier. We believe advancements in diagnostic tools and management guidelines could explain this. This also supports the rising shift from invasive to conservative and minimally invasive options when managing different surgical conditions.

## Conclusions

In conclusion, the management of uncomplicated appendicitis is still a debatable topic. Considering the current studies' findings, conservative treatment illustrates a cost-effective option with lower complication rates that could be utilised by considering the presence or absence of certain clinical, laboratory and imaging findings that favour surgical treatment, which ultimately holds a higher efficacy rate. Thus, the decision-making process is highly case-based, aiming to enhance treatment efficacy, lower complication rate and reduce costs wherever possible. Additionally, to aid this process making in the future, we recommend that future research include the number of elective and emergency appendectomies done after appendicitis recurrence following conservative management and include a more detailed cost-analysis of both treatment options.
